# Investigation and Evaluation of Genetic Diversity of *Plasmodium falciparum* Kelch 13 Polymorphisms Imported From Southeast Asia and Africa in Southern China

**DOI:** 10.3389/fpubh.2019.00095

**Published:** 2019-04-24

**Authors:** Jun Feng, Xiangli Kong, Dongmei Xu, He Yan, Hongning Zhou, Hong Tu, Kangming Lin

**Affiliations:** ^1^Chinese Center for Disease Control and Prevention, National Institute of Parasitic Diseases, Shanghai, China; ^2^Key Laboratory of Parasite and Vector Biology, Ministry of Health, Shanghai, China; ^3^World Health Organization Collaborating Centre for Tropical Diseases, Shanghai, China; ^4^National Center for International Research on Tropical Diseases, Shanghai, China; ^5^Shandong Institute of Parasitic Diseases, Shandong Academy of Medical Sciences, Jining, China; ^6^Department of Food and Pharmaceutical Engineering, Shijiazhuang University of Applied Technology, Shijiazhuang, China; ^7^Yunnan Institute of Parasitic Diseases, Pu'er, China; ^8^Guangxi Zhuang Autonomous Region Center for Disease Control and Prevention, Institute of Parasitic Diseases, Nanning, China

**Keywords:** malaria, *Plasmodium falciparum*, artemisinin resistance, Kelch 13, microsatellite

## Abstract

**Objectives:** In this study, we aimed to analyse the genetic diversity Kelch 13 (K13) propeller allele of the *Plasmodium falciparum* isolates mainly imported from Southeast Asia and Africa in southern China, including the provinces of Yunnan and Guangxi.

**Methods:** At enrolment, we collected blood samples from patients with confirmed cases of malaria infection between January 2012 and December 2017, for analysis. Individual patient information was obtained via a malaria surveillance system. The malaria infections and *P. falciparum* K13 mutations were diagnosed by using a nested polymerase chain reaction (PCR) method.

**Results:** The K13 mutations were identified in 283 *P. falciparum* isolates from 18 counties in Yunnan and 22 counties in Guangxi. Of Forty-six isolates (46/283, 16.3%) that harbored K13 mutant alleles were detected: 26.8% in Yunnan (33/123) and 8.1% in Guangxi (13/160). A total of 18 different K13 mutations were detected. Only the F446I mutation was detected in Yunnan isolates, and F446I was more frequent (20/46, 43.5%) than other alleles. Further, the temporal distribution of the F446I mutation ratio from 2012 to 2015 exhibited no significant difference in Yunnan Province (2012, 2/13, 15.4%; 2013, 7/40, 17.5%; 2014, 7/33, 21.2%; 2015, 4/37, 10.8%, *p* = 0.121). A578S allele was the main K13 mutation (5/283, 1.8%) from Africa. The K13 mutants were present in 33.3% of indigenous isolates, 27.4% of isolates from Southeast Asia, and 7.9% of isolates from Africa. The analysis of 10 neutral microsatellite loci of 60 isolates showed that at the TAA109 locus, the expected heterozygosity of F446I (*H*_*e*_ = 0.112 ± 0.007) was much lower than that of wild type and other mutation types in Myanmar isolates. With respect to geographic distribution, TAA109 also exhibited a significant difference between isolates from Southeast Asia (*H*_*e*_ = 0.139 ± 0.012) and those from Africa (*H*_*e*_ = 0.603 ± 0.044).

**Conclusions:** The present findings on the geographic diversity of K13 mutant alleles in *P. falciparum* may provide a basis for routine molecular surveillance and risk assessment, to monitor artemisinin resistance (ART) in China. Our results will be helpful for enriching the artemisinin resistance database in China during the elimination and post-elimination phases.

## Introduction

Artemisinin-based combination therapies (ACTs) have been recommended as first-line drugs for the treatment of *Plasmodium falciparum* malaria infection in endemic regions by the World Health Organization (WHO). ACTs have contributed to a sharp reduction of the malaria burden in many countries (1). However, artemisinin resistance (ART), initially detected near the Thai–Cambodia border has spread across the Greater Mekong Subregion (GMS) and been confirmed in five countries (Myanmar, Cambodia, Laos, Vietnam, and Thailand) of the GMS (2, 3). Therefore, the WHO have launched the Global Plan for Artemisinin Resistance Containment to contain ART-resistant isolates (4). ART and its derivatives have been widely used in China for nearly three decades (5). For example, Yunnan Province has a long history of ART monotherapy, and *in vitro* studies have shown reduced susceptibility of *P. falciparum* from the region to ART (6). It would be catastrophic if ART-resistant isolates have spread across the GMS to the border areas of China, especially to Yunnan and Guangxi provinces in southern China (7, 8). Such spread is of great concern for malaria elimination in China. Furthermore, *P. falciparum* has recently exhibited a sharp increase in China, particularly in Yunnan and Guangxi provinces (9). Besides, Yunnan is one of the most severe endemic regions in China and most cases of *P. falciparum* malaria are imported from Southeast Asia (10).

The *PfKelch* 13 propeller (K13) has been identified as a molecular marker for ART-resistant isolates. More than 5% of surveyed patients carrying K13 resistance-confirmed mutations, and all of whom have been found to have either persistent parasitaemia by microscopy on day 3 or a half-life of the parasite clearance slope ≥ 5 h after treatment are considered as confirmed endemic artemsinin resistance (11). A total of 186 K13 alleles have been identified and five of them (C580Y, R539T, Y493H, N458Y, and I543T) have been confirmed as ART-resistant isolates (11). Furthermore, delayed clearance following ART treatment has been detected around the China–Myanmar border, with F446I identified as the predominant K13 mutant allele (7, 12). In central China, no K13 resistance alleles have been validated, except in Henan Province where R539T and P574L have been identified as the main alleles found among migrant workers returning from African countries (13, 14). Recently, a patient who returned from Equatorial Guinea to Jiangsu Province was identified as harboring M579I, which has been linked to ART resistance with a 2.29% *in vitro* survival rate by ring-stage survival assay (15). Those findings provide basic molecular surveillance data regarding the potential emergence of ART-resistant isolates in African countries. However, little is known about differences in the distribution and genetic diversity of the K13 propeller allele between locally acquired and imported samples, such as from Southeast Asia and Africa. Therefore, understanding the attributes of the K13 allele in ART-resistant isolates and its genetic diversity in China is of great importance, especially for areas bordering Myanmar and those with many malaria cases imported from African countries, to determine whether ART resistance is emerging or has already spread to China.

In this study, we aimed to investigate and evaluate the genetic diversity of the K13 propeller allele in Yunnan and Guangxi provinces in southern China, which have the greatest number of malaria cases imported from Southeast Asia and Africa, respectively. We sought to reveal the K13 mutation status and monitor the potential emergence of ART resistance in China.

## Materials and Methods

### Study Design

In this study, an indigenous case refers to malaria acquired by mosquito transmission in China. The imported case refers to the patient who acquired the illness from a known malaria-prevalent area outside China (16).

### Study Sites and Samples

A total of 285 *P. falciparum*-infected blood samples were examined in this study. The individual data were obtained during 2012–2017 via a malaria surveillance system of the National Institute of Parasitic Diseases, Chinese Center for Diseases Control and Prevention. The study was conducted in Yunnan Province and Guangxi Zhuang Autonomous Region. Yunnan has a population of 47 million people living in its 16 prefectures and 129 counties. Among them, 18 counties bordering Myanmar were included in this study. Because no cases of *P. falciparum* infection were reported in Gongshan and Fugong counties; therefore, Lianghe and Jiangcheng counties were included instead because these are adjacent to border counties. For each year from 2012 to 2017, the annual reported malaria incidence rate in Yunnan Province was 0.18, 0.12, 0.11, 0.13, 0.07, and 0.05 per 10,000 residents, respectively. Guangxi is a province with some of the highest numbers of imported cases reported in China. The last indigenous case in Guangxi was reported in 2012; all subsequent cases have been imported from abroad, particularly from African countries. In 2013, owing to clusters of migrant workers returning from Ghana to Shanglin County in Guangxi, the number of imported malaria cases rose substantially, contributing greatly to the number of reported malaria cases nationwide in that year (17).

At enrolment, we collected blood samples from patients with a confirmed malaria diagnosis by microscopy, polymerase chain reaction (PCR) (18), or rapid diagnostic tests (Malaria Pf/pan, Wondfo Biotech Co., LTD., Guangzhou, China) in the laboratory in Yunnan and Guangxi provinces, from January 2012 to December 2017. Approximately 200 μL of finger-prick blood was spotted onto Whatman 3MM filter paper (GE Healthcare, Boston, MA, USA) and air dried. Each sample was labeled with the study number, name, and date and stored at −20°C until DNA extraction.

All individual cases included in this study from the web-based reporting system (China Information System for Diseases Control and Prevention) were carefully reviewed and analyzed.

### Preparation of DNA Template, Nested PCR, and Sequencing

Parasite genomic DNA from all blood-spot samples collected into microcentrifuge tubes and one filter spot (diameter 5 mm) was extracted using a QIAamp DNA Blood Mini Kit (QIAGEN, Valencia, CA), 500 μL elution buffer was added into each tube by following the dried blood spots protocol for the kit. The K13 propeller gene (*PF3D7_1343700*) was amplified using a nested PCR method as described previously (2). PCR products were separated and visualized using 2% agarose gel electrophoresis and GelRed (Shanghai Aberlong Biological Co., Ltd., Shanghai, China). The size of K13 fragment was 849 bp covering mutation sites from 427 amino acids to 709 amino acids. Sequences were analyzed using the BLAST website (http://blast.ncbi.nlm.nih.gov/). Multiple nucleotide sequence alignment and analysis were performed using the DNAMAN sequence alignment editor (https://www.lynnon.com/pc/framepc.html).

### Microsatellite Loci Genotyping

Ten neutral microsatellite markers, including ARA2 (Chr11), TA1 (Chr6), TAA42 (Chr5), TAA60 (Chr13), TAA81 (Chr5), TAA87 (Chr6), TAA109 (Chr6), pfPK2 (Chr12), Polyα (Chr4), and B5M2 (Chr7) were selected for analysis (19). The first nine loci have been previously used as putatively neutral microsatellite markers for population genetic studies (20). The protocol followed has been previously described (21), and the size of the amplification products was determined using capillary electrophoresis assay. For each isolate, minor peaks more than one-third the height of the predominant allele for each locus were considered to indicate multiple alleles per locus (20).

### Population Genetic Analysis

Population genetics were preferably assessed only in samples with single infections because the use of samples with multiple infections could result in bias. To measure the genetic diversity, the number of haplotypes (h), number of different alleles (N_a_), number of effective alleles (N_e_), number of private alleles (N_p_), and the expected heterozygosity (*H*_*e*_) were evaluated using the GenAlEx 6 microsatellite plugin (22). *H*_*e*_ was calculated using the following formula:

*H*_*e*_ = [n/(n−1)] (1–∑${\text{p}}_{\text{i}}^{2}$)], and 2(n−1)/n^3^{2(n−2)[∑(${\text{p}}_{\text{i}}^{3}$-(∑${\text{p}}_{\text{i}}^{2}$)^2^]} for sampling variance, where n is the number of sampled infections and p_i_ is the frequency of the ith allele.

### Statistical Analyses

Data were analyzed using Microsoft Excel 2003 and SAS version 9.2 (SAS Institute Inc., Cary, NC, USA). The map was created by ArcGIS 10.1 (Environmental Systems Research Institute, Inc., Redlands, CA, USA). The Fisher's exact test was used to assess the differences in gene polymorphisms between indigenous cases and imported cases from abroad, as well as the temporal distribution of mutant alleles. Calculated *p*-values were considered statistically significant with *p* < 0.05.

### Ethical Considerations

The study was reviewed and approved by the ethical committee of the National Institute of Parasitic Diseases, Chinese Center for Disease Control and Prevention (NIPD, China CDC). This study was carried out in accordance with the recommendations of Chinese National Antimalarial Resistance Surveillance Plan, China CDC. The protocol was approved by the NIPD, China CDC. All subjects gave written informed consent in accordance with the Declaration of Helsinki.

## Results

### Study Samples

**Figure 1:**
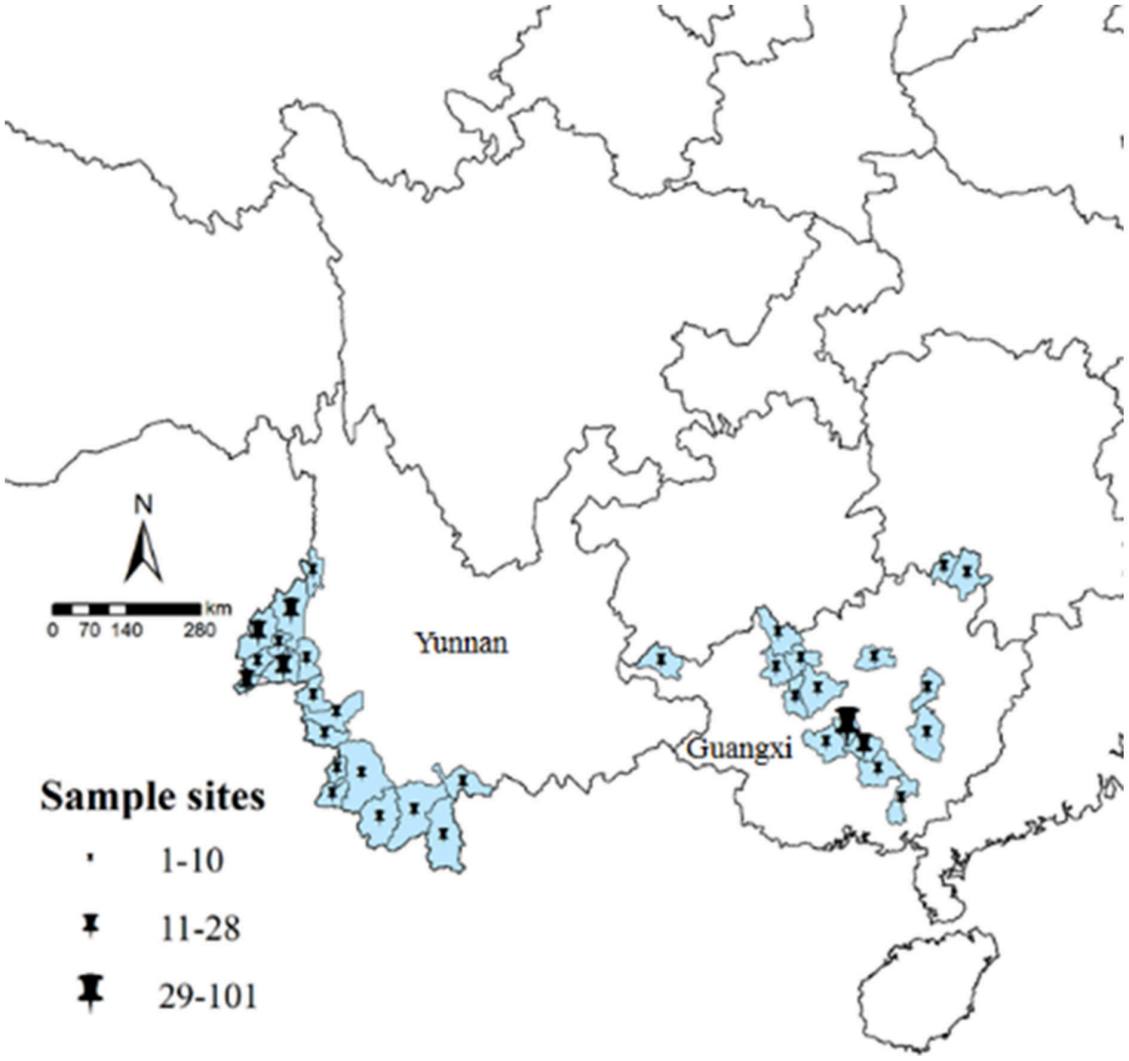
Study sample collection sites in Yunnan and Guangxi. All counties are labeled according to the number of obtained samples, using ArcGIS 10.1.

A total of 285 isolates were collected: 123 isolates were collected from 18 counties in Yunnan, including 6 indigenous isolates and 117 imported isolates from Southeast Asia (116 isolates from Myanmar and 1 isolate from Laos). Another 162 isolates were collected from Guangxi, all of which were imported from African countries. Because 2 isolates were unable to be sequenced, therefore 283 effective isolates were finally obtained for analysis (Figure 1). All the 283 isolates were confirmed as positive by using three methods (microscopy plus RDTs plus PCR), the PCR gel picture were showed in Figure S1.

### Epidemiology

Isolates from Yunnan were mainly distributed among the counties of Yingjiang (*n* = 28, 22.8%), Ruili (*n* = 23, 18.7%), and Tengchong (*n* = 21, 17.1%). Most isolates were from male patients (*n* = 111, 90.2%) with the age of 16–35 years (*n* = 108, 87.8%). The isolates were mainly collected in 2013 (*n* = 40, 32.5%). Isolates from Guangxi were mainly from the counties of Shanglin (*n* = 101, 63.1%), Bingyang (*n* = 14, 8.8%), and Nanning (*n* = 12, 7.5%). Similar to Yunnan, the isolates were mainly from patients aged 16–59 years (*n* = 158, 98.8%) and most isolates were collected in 2013 (*n* = 53, 33.1%) (Table S1).

### K13 Propeller Genetic Diversity

To investigate genetic diversity in K13 propeller allele, all 283 *P. falciparum* isolates were assayed and sequenced. K13 mutations were detected in 46 isolates (16.3%), with 33 of these from Yunnan (26.8%, 33/123) and 13 from Guangxi (8.1%, 13/160) (Table 1). Two local *P. falciparum* isolates harboring F446I mutant alleles were reported in Yunnan (Nabang in Yingjiang County and Zhefang in Manshi County); the remaining 44 isolates were imported from abroad.

**Table 1:** Prevalence of *Plasmodium falciparum* K13 mutant alleles in Yunnan and Guangxi, 2012–2017.

**Province**	**Year**	**No. of samples**	**Mutation (%)**	**Indigenous cases**	**Mutation (%)**	**Imported cases**	**Mutation (%)**	***p***
Yunnan	2012	13	3 (23.1)	1	1 (100)	12	2 (16.7)	*p* < 0.05
	2013	40	11 (27.5)	4	0 (0)	36	11 (30.6)	NS
	2014	33	12 (36.4)	1	1 (100)	32	11 (34.4)	*p* < 0.05
	2015	37	7 (18.9)	0	0	37	7 (18.9)	NS
	Subtotal	123	33 (26.8)	6	2 (33.3)	117	31 (26.5)	*p =* 0.001
Guangxi	2012	11	1 (11.1)	0	0	11	1 (11.1)	NS
	2013	53	3 (5.7)	0	0	53	3 (5.7)	NS
	2014	20	3 (15.0)	0	0	20	3 (15.0)	NS
	2015	14	1 (7.1)	0	0	14	1 (7.1)	NS
	2016	32	2 (6.2)	0	0	32	2 (6.2)	NS
	2017	30	3 (10.0)	0	0	30	3 (10.0)	NS
	Subtotal	160	13 (8.1)	0	0	160	13 (8.1)	*p* < 0.05
Total		283	46 (16.3)	6	2 (33.3)	277	44 (15.9)	*p* < 0.05

*NS, not significant*.

**Figure 2:**
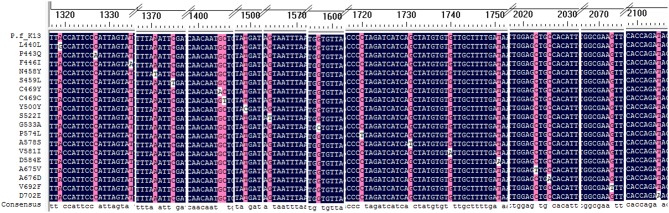
Alignment of all mutation sites of K13 listed in the study. All 18 K13 mutation sites were alignment with *Plasmodium falciparum* 3D7 kelch gene.

A total of 18 different K13 mutant alleles were observed, including 15 non-synonymous mutation sites and 3 synonymous mutation sites (L440L, C469C, and Y500Y) (Figure 2). F446I was the more frequently detected allele (20/46, 43.5%) than other mutation alleles. A total 18 isolates harboring the F446I allele were found in patients returning from Myanmar and 2 were found in patients from China (*p* < 0.05). Additionally, N458Y, which is associated with ring-stage survival assay, was found in two samples from Myanmar. The mutant allele A578S was detected in 6 patients, 5 from Ghana and 1 from Myanmar (*p* < 0.05). Further, three isolates harboring the A676D allele were all found in patients returning from Myanmar (Table 2).

**Table 2:** Prevalence of genotype candidate K13 mutant alleles of indigenous (China), Southeast Asian, and African isolates.

**K13 mutation**	**Indigenous**		**Southeast Asia**		**Africa**		**Subtotal**
	***N***	**Prevalence (%)**	***N***	**Prevalence (%)**	***N***	**Prevalence (%)**	
L440L	0	0	0	0	1	0.6	1
P443Q	0	0	1	0.9	0	0	1
F446I	2	33.3	18	15.7	0	0	20
N458Y	0	0	2	1.7	0	0	2
S459L	0	0	1	0.9	0	0	1
C469Y	0	0	1	0.9	0	0	1
C469C	0	0	0	0	2	1.2	2
Y500Y	0	0	0	0	1	0.6	1
S522I	0	0	0	0	1	0.6	1
G533A	0	0	1	0.9	0	0	1
P574L	0	0	1	0.9	0	0	1
A578S	0	0	1	0.9	5	3.1	6
V581I	0	0	1	0.9	0	0	1
D584E	0	0	0	0	1	0.6	1
A675V	0	0	0	0	1	0.6	1
A676D	0	0	3	2.6	0	0	3
V692F	0	0	1	0.9	0	0	1
D702E	0	0	0	0	1	0.6	1
Total	2	33.3 (2/6)	31	27.0 (31/115)	13	8.0 (13/162)	46

From 2012 to 2015, the temporal distribution of the F446I mutation rate exhibited no significant difference (2012, 2/13, 15.4%; 2013, 7/40, 17.5%; 2014, 7/33, 21.2%; 2015, 4/37, 10.8%, rang from 10.8 to 21.2%, *p* = 0.121).

### K13 Propeller Geographic Differentiation

For local *P. falciparum* isolates, 2 of 6 isolates were found to have mutated sites (33.3%), and all were F446I. Furthermore, 31 of 115 isolates from countries in Southeast Asia exhibited K13 mutations (27.0%). Conversely, only 13 of 160 isolates from Africa harbored mutated sites (8.1%) (*p* < 0.05). With respect to the distribution of K13 mutant alleles in China, 33 isolates harboring mutated alleles were observed in Yunnan, mainly in Tengchong (14/33, 42.4%), Ruili (9/33, 27.3%), and Yingjiang (3/33, 9.1%) counties. In Guangxi, 13 isolates harbored K13 mutated alleles were mainly from Ghana (9/13, 69.2%), and there was one patient each from Sierra Leone, Cameroon, the Congo, and Equatorial Guinea (Table 3).

**Table 3:** Distribution of the K13 mutant allele in Yunnan and Guangxi provinces, with different imported sources.

**Province**	**County**	**K13 mutation**			**Year**	**Source**
		***N***		**Codon position**		
Yunnan	Tengchong	14	1	P574L	2012	Myanmar
			1	P443Q	2013	Myanmar
			2	F446I	2013	Myanmar
			1	G533A	2013	Myanmar
			1	A578S	2013	Myanmar
			1	A676D	2014	Myanmar
			1	C469Y	2014	Myanmar
			2	N458Y	2014	Myanmar
			2	F446I	2014	Myanmar
			2	F446I	2015	Myanmar
	Ruili	9	3	F446I	2013	Myanmar
			2	F446I	2014	Myanmar
			1	S459L	2014	Myanmar
			1	A676D	2014	Myanmar
			1	F446I	2015	Myanmar
			1	A676D	2015	Myanmar
	Yingjiang	3	1	F446I	2013	Myanmar
			1	F446I	2014	Myanmar
			1	F446I	2014	Indigenous (Nabang)
	Mangshi	3	1	F446I	2012	Indigenous (Zhefang)
			1	F446I	2012	Myanmar
			1	V581I	2015	Laos
	Menglian	1	1	F446I	2013	Myanmar
	Longchuan	1	1	V692F	2013	Myanmar
	Lushui	1	1	F446I	2014	Myanmar
	Jinghong	1	1	F446I	2015	Myanmar
Guangxi	Shanglin	8	3	A578S	2013	Ghana
			1	A578S	2015	Ghana
			1	A578S	2017	Ghana
			1	C469C	2016	Congo
			1	A675V	2015	Ghana
			1	D702E	2016	Ghana
	Liucheng	1	1	S522I	2015	Sierra Leone
	Donglan	1	1	D584E	2016	Ghana
	Liuzhou	1	1	Y500Y	2017	Equatorial Guinea
	Binyang	1	1	C469C	2017	Cameroon
	Nanning	1	1	L440L	2017	Ghana

### Microsatellite Analysis

We genotyped 60 isolates (including 17 from African countries and 43 from Southeast Asia) using analysis of 10 neutral microsatellite loci. According to the K13 single nucleotide polymorphism (SNP) sequencing data and sample size, and because F446I is the prominent mutant allele in the China–Myanmar border area, the microsatellite loci data were classified into three types: wild type (*n* = 16), F446I allele type (*n* = 20), and other mutant allele types (MT, *n* = 24). Generally, no significant difference in allele frequency was observed between wild type and MT; the *H*_*e*_ value of F446I was lower than that of wild type and MT (Table S2). Since most of the F446I were imported from Myanmar, herein we found the F446I type at TAA109 locus (*H*_*e*_ = 0.112 ± 0.007) had fewer alleles than wild type (*H*_*e*_ = 0.385 ± 0.028) and MT (*H*_*e*_ = 0.478 ± 0.036) in Myanmar isolates (Table 4). With respect to geographic distribution, TAA109 exhibited a significant difference between isolates from Southeast Asia (*H*_*e*_ = 0.139 ± 0.012) and those from African countries (*H*_*e*_ = 0.603 ± 0.044) (Table 5).

**Table 4:** Microsatellite analysis for the analyzed isolates in Myanmar.

**Locus**	**WT**	**MT**	**F446I**
ARA2	0.819	0.707	0.563
B5M2	0.752	0.732	0.695
pfg377	0.362	0.304	0.620
pfpk2	0.819	0.815	0.647
polya	0.867	0.862	0.847
TA1	0.790	0.634	0.679
TAA42	0.590	0.728	0.611
TAA60	0.810	0.754	0.789
TAA81	0.886	0.779	0.795
TAA87	0.867	0.866	0.789
TAA109	0.385	0.478	0.112

*Isolates classified into K13 mutation types as wild type (WT), F446I, and other mutation types (MT)*.

**Table 5:** Microsatellite analysis for the analyzed isolates which were classified according to imported source as Southeast Asia and Africa; indigenous isolates are included in Southeast Asia isolates.

**Locus**	**Southeast Asia**	**Africa**
ARA2	0.627	0.647
B5M2	0.724	0.699
pfpk2	0.748	0.853
polya	0.868	0.890
TA1	0.624	0.772
TAA42	0.614	0.728
TAA60	0.829	0.743
TAA81	0.846	0.684
TAA87	0.850	0.868
TAA109	0.139	0.603

## Discussion

There are two great challenges facing the elimination of malaria in China. One is the presence of cross-border malaria along the China–Myanmar border in Yunnan Province. Even though local transmission has been interrupted, with no indigenous cases occurring in 2017, reintroduction may occur as the *Anopheles* mosquito remains present in the region (9). The other challenge is the increasing number of imported cases, with migrant populations arriving frequently from endemic countries or regions, such as Africa, which makes case management and surveillance very difficult. It is known that migrant populations are at risk of exposure to malaria and these groups are considered to carry and spread resistant parasites (23). In this study, we selected Yunnan and Guangxi, the two provinces in China with the highest number of malaria cases imported from Southeast Asia and Africa, respectively, to understand the genetic diversity of K13 allele polymorphisms and conduct population genetic analysis using microsatellite loci genotyping.

Our findings provide evidence that areas near the border between China and northern Myanmar have a high prevalence of the F446I mutation in the K13 gene, which is similar to other previous reports (7, 24–26). To date, F446I has mostly been prevalent near the China–Myanmar and Myanmar–India borders. Near the China–Myanmar border, F446I has been associated with delayed parasite clearance (12); however, this is not relevant to treatment outcomes in the Myanmar–India border area (27). In addition, A676D was previously identified in three studies including samples from northern Myanmar (26, 28, 29). N458Y is associated with ring-stage survival assay values >1% and has been proved to be a molecular marker associated with ART-resistant isolates previously reported in Cambodia and Myanmar (3, 25, 30). Other candidates associated with ART-resistant isolates according to the WHO (11), including Y493H, R539T, I543T, R561H, and C580Y, were not detected in this study. Interestingly, P574L, the most widespread mutation distributed in northeast Myanmar, was seen at a relatively low prevalence in neighboring Ruili County of Yunnan. Thus, few of the K13 mutants currently in Yunnan have a high prevalence in Cambodia or in southern and central Myanmar. Therefore, the present mutations indicate that the China–Myanmar border region, exhibits a distribution pattern of K13 mutant alleles that differs from other regions in the GMS (31).

In general, there was a relatively low prevalence of K13 mutations among samples from Africa in this study. A578S had a higher prevalence than other mutant alleles in the African samples. This mutation has been reported as having no correlation with ART-resistant isolates in previous studies and is a K13 polymorphism commonly detected throughout Africa, suggesting that A578S possibly has a distinct origin and evolution pathway (32). However, the A578S mutation was detected in an isolate from one of our study participants who had returned from Myitkyina, a city located in northern Myanmar. S522I and A675V were found in only 1 isolate from Sierra Leone and Ghana, respectively, which may reveal that these mutations originated in Africa (33, 34). Apart from this, the samples from Africa exhibited a high prevalence of synonymous mutation sites; except for Y500Y, three had been previously reported (35), and the other two mutations were newly detected.

In K13 population genetic analysis, we conducted a microsatellite assay to assess and evaluate geographic evolution. F446I was seen to exhibit a lower *H*_*e*_ value than wild type and MT alleles from Yunnan Province and northern Myanmar, suggesting that this mutant allele evolved locally in the region (Table S2). Thus, F446I could be selected and adopted for use as a molecular marker in the China–Myanmar border region, to identify the geographic origins and dissemination of ART-resistant *P. falciparum*. However, further *in vivo* and *in vitro* studies are needed, such as clinical efficacy surveillance associated with F446I mutations. Interestingly, the TAA109 locus, showed varying *H*_*e*_ values among F446I, wild type, and MT, as well as diversity between isolates from Southeast Asia and Africa. This highlights the need for further research to understand the relationship between TAA109 and the F446I mutation, such as genome-wide sequencing and linkage disequilibrium analysis. We propose that ART resistance is unlike resistance to chloroquine and sulfadoxine-pyrimethamine in that the latter are associated with relatively fixed mutations in codons. For example, isolates from Africa exhibit the highly resistant *pfcrt* (C_72_V_73_I_74_E_75_T_76_) and *dhfr* (*P. falciparum* dihydrofolate reductase gene, C_50_I_51_R_59_N_108_I_164_) alleles (36, 37). The mechanism of K13 candidates associated with ART-resistant *P. falciparum* could be a multigenetic process and could also be combined with other molecular markers, such as *pfmdr1, pfmdr2, pfatpse* 6, and *pfarps 10*, all of which have been previously reported to be related to ART resistance (38–40). The next work will carry out msp1 and msp2 PCR genotyping of samples used in microsatellite analysis, to explore the further reason of the low diversity at TAA109 which may due to the selective sweep at K13 locus.

For the limitation, of the malaria cases studied, only six cases were indigenous malaria. Considering that we intended to analyse the diversity of locally acquired infections and potential evolution of ART resistance in China, the number of samples were not adequate.

In summary, the present data indicated a high prevalence of the F446I mutant allele, detected among both indigenous and imported malaria cases from Myanmar, especially for counties along the China–Myanmar border. In addition, a distribution pattern of K13 mutant alleles was observed that differed from other regions of the GMS or Africa. Microsatellite analysis indicated that the F446I allele had a much lower *H*_*e*_ value than both wild type and MT at locus TAA109. As for imported cases from Africa, few K13 mutant alleles related to ART-resistance were found, with A578S predominating in African samples. Routine molecular surveillance and risk assessment should be conducted to monitor ART resistance throughout the study region. The present data will be helpful in development of a genetic molecular marker to trace malaria infection sources during the malaria elimination and post-elimination phases, to achieve the goal of eliminating ART resistance in *P. falciparum* throughout the GMS.

## Author Contributions

JF designed and conducted the study, and drafted the article. XK and HT carried out the statistical analysis. HY carried out the molecular experiment. DX carried out the epidemiology survey and recording. HZ and KL supported the study and performed sample collection.

### Conflict of Interest Statement

The authors declare that the research was conducted in the absence of any commercial or financial relationships that could be construed as a potential conflict of interest.
