# Supplementary material for: Investigation and Evaluation of Genetic Diversity of Plasmodium falciparum Kelch 13 Polymorphisms Imported From Southeast Asia and Africa in Southern China
Source: Front Public Health. 2019 Apr 24;7:95. doi: 10.3389/fpubh.2019.00095 (PMC6491575; doi:10.3389/fpubh.2019.00095)
Supplement: Supplementary file 1 [file Data_Sheet_1.pdf]

Supplemental Materials

Table S1The geographical, temporal, age and gender distribution for the studied samples.

Province	County	No. of samples	Male					Female					Year					
			≤15	16-35	36-59	≥60	Subtotal	≤15	16-35	36-59	≥60	Subtotal	2012	2013	2014	2015	2016	2017
Yunnan[a]	Yingjiang	28	0	13	11	0	24	2	2	0	0	4	2	5	12	9	0	0
	Ruili	23	3	12	5	0	20	1	2	0	0	3	1	9	6	7	0	0
	Tengchong	21	0	10	9	0	19	0	1	1	0	2	1	8	7	5	0	0
	Mangshi	13	0	8	3	0	11	0	1	1	0	2	5	4	1	3	0	0
	Canyuan	8	0	3	4	0	7	0	0	1	0	1	3	4	1	0	0	0
	Genma	4	0	4	0	0	4	0	0	0	0	0	0	2	2	0	0	0
	Menglian	4	0	1	3	0	4	0	0	0	0	0	0	1	0	3	0	0
	Longling	3	0	1	1	1	3	0	0	0	0	0	0	2	0	1	0	0
	Longchuan	3	0	1	2	0	3	0	0	0	0	0	0	2	0	1	0	0
	Lushui	3	1	2	0	0	3	0	0	0	0	0	0	0	1	2	0	0
	Jinghong	2	0	1	1	0	2	0	0	0	0	0	0	0	1	1	0	0
	Lancang	2	0	2	0	0	2	0	0	0	0	0	0	0	0	2	0	0
	Lianghe	2	0	0	2	0	2	0	0	0	0	0	0	0	1	1	0	0
	Ximen	2	0	1	1	0	2	0	0	0	0	0	1	1	0	0	0	0
	Zhengkang	2	0	2	0	0	2	0	0	0	0	0	0	0	1	1	0	0
	Jiangcheng	1	0	0	1	0	1	0	0	0	0	0	0	0	0	1	0	0
	Menghai	1	0	0	1	0	1	0	0	0	0	0	0	1	0	0	0	0
	Mengla	1	0	1	0	0	1	0	0	0	0	0	0	1	0	0	0	0
	Subtotal	123	4	62	44	1	111	3	6	3	0	12	13	40	33	37	0	0
Guangxi	Shanglin	101	0	44	50	2	96	0	3	2	0	5	7	44	13	10	12	15
	Bingyang	14	0	6	8	0	14	0	0	0	0	0	1	3	3	3	3	1

Nanning	12	0	4	5	0	9	0	1	2	0	3	2	3	1	0	2	4
Dahua	10	0	4	6	0	10	0	0	0	0	0	1	3	2	0	3	1
Liucheng	3	0	1	2	0	3	0	0	0	0	0	0	0	1	1	1	0
Liuzhou	3	0	0	2	0	2	0	1	0	0	1	0	0	0	0	1	2
Jinxiu	2	0	0	2	0	2	0	0	0	0	0	0	0	0	0	0	2
Longlin	2	0	0	2	0	2	0	0	0	0	0	0	0	0	0	1	1
Donglan	1	0	0	1	0	1	0	0	0	0	0	0	0	0	0	1	0
Douan	1	0	0	0	0	0	0	1	0	0	1	0	0	0	0	0	1
Guigang	1	0	1	0	0	1	0	0	0	0	0	0	0	0	0	1	0
Guiping	1	0	0	0	0	0	0	1	0	0	1	0	0	0	0	1	0
Hechi	1	0	0	1	0	1	0	0	0	0	0	0	0	0	0	1	0
Hengxian	1	0	0	1	0	1	0	0	0	0	0	0	0	0	0	1	0
Nandang	1	0	1	0	0	1	0	0	0	0	0	0	0	0	0	1	0
Pubei	1	0	0	1	0	1	0	0	0	0	0	0	0	0	0	1	0
Quanzhou	1	0	0	1	0	1	0	0	0	0	0	0	0	0	0	0	1
Wuming	1	0	0	1	0	1	0	0	0	0	0	0	0	0	0	1	0
Xiangshan	1	0	1	0	0	1	0	0	0	0	0	0	0	0	0	0	1
Yulin	1	0	1	0	0	1	0	0	0	0	0	0	0	0	0	0	1
Ziyuan	1	0	0	1	0	1	0	0	0	0	0	0	0	0	0	1	0
Subtotal	160	0	63	84	2	149	0	7	4	0	11	11	53	20	14	32	30
Total	283	4	125	128	3	260	3	13	7	0	23	24	93	53	51	32	30

[a] A total of 18 counties in Yunnan Province neigbouring Myanmar, however, since no P. falciparum cases were reported by counties of Gongshan and Fugong, and counties of Lianghe and Jiangcheng were also neighbouring with border counties, therefore Lianghe and Jiangcheng were also included in this study.

Table S2 The 10 neutral microsatellite analysis for the isolates from indigenous, Southeast Asia (Myanmar and Laos) and Africa, 2012-2017.

Code	Year	Source	K13 sites	ARA2	B5M2	pfpk2	polya	TA1	TAA-42	TAA60	TAA81	TAA87	TAA109
1	2013	Indigenous	WT	57.6	159.7	166.5	174.8	167	183.8	184.3	125.3	86.1	160
2	2014	Indigenous	F446I	56	160	166.6	166.3	163.8	180.2	184.1	125.1	95.2	321.9
3	2012	Myanmar	WT	76.1	159.7	166.5	157.3	163.9	180	198.4	116.2	107	159.8
4	2012	Myanmar	WT	74.3	147.6	163.5	174.8	164	180.1	198.2	134.1	89.2	159.8
5	2012	Myanmar	WT	67.4	141.4	160.5	157.3	164.1	183.7	200.2	122.3	104	171.7
6	2013	Myanmar	WT	66.1	159.8	172.5	157.3	181.9	183.8	200	119.3	86.1	159.8
7	2013	Myanmar	WT	73.5	147.6	160.6	162.6	163.9	180.1	194.1	125.2	104.2	159.9
8	2014	Myanmar	WT	70.4	141.6	175.6	162.6	163.9	180.2	198.2	134.1	104.1	165.9
9	2014	Myanmar	WT	67.8	141.5	166.6	187.9	158	184.3	198.2	113.1	107.2	159.9
10	2014	Myanmar	WT	68	141.5	166.4	187.8	178.3	180.1	192.2	116.2	110.1	159.8
11	2016	Myanmar	WT	65.5	141.7	166.6	174.7	167	180.3	184.2	110.2	95.2	159.7
12	2012	Myanmar	F446I	66.1	160	166.5	160.4	158	183.9	188.2	116.2	95	160
13	2012	Myanmar	F446I	65.7	159.2	163.6	165.6	164	183.6	198.1	125.2	113.1	159.8
14	2013	Myanmar	F446I	75.8	184.5	166.6	160.4	161.5	180.2	200.1	110.1	104.2	159.8
15	2013	Myanmar	F446I	73.8	141.4	163.6	165.6	166.1	184.2	194.5	113.1	113.2	159.6
16	2013	Myanmar	F446I	66.2	147.7	166.5	163.4	164	187.9	192.3	110.3	98	159.9
17	2013	Myanmar	F446I	68.1	159	163.5	157.3	163.9	180.2	188.1	125.2	104.2	159.8
18	2013	Myanmar	F446I	66.3	184.3	163.6	162.6	155.2	180	183.8	116.1	104.1	159.8
19	2013	Myanmar	F446I	67.5	141.5	163.5	157.4	166.9	184.2	183.9	116.2	107.3	159.7
20	2013	Myanmar	F446I	67.4	160	172.5	160.5	166.8	180.1	184	125.1	95.1	160.1
21	2014	Myanmar	F446I	73.7	141.4	166.5	163.4	166.8	180.1	198.2	113.2	104.1	190.1
22	2014	Myanmar	F446I	66.5	160	163.5	163.5	156.2	180.1	192.1	125.3	104.3	159.8

23	2014	Myanmar	F446I	67.1	160	166.6	187.9	163.9	183.7	198.2	116.2	107.2	159.9
24	2014	Myanmar	F446I	74.8	141.7	163.5	135.8	166.1	186.5	192	125.1	89.1	159.8
25	2014	Myanmar	F446I	76.2	141.5	166.5	160.4	163.8	180.1	192.3	125.1	95.1	160.2
26	2014	Myanmar	F446I	66.5	159.7	172.5	160.3	163.9	180.2	184.1	113.2	95.2	159.8
27	2015	Myanmar	F446I	67.1	160	166.5	165.5	163.8	183.7	184.2	134.2	95.2	159.9
28	2015	Myanmar	F446I	64.8	159.7	166.6	157.4	164	180	192.1	116.1	104.2	160.1
29	2015	Myanmar	F446I	65.2	141.7	166.6	157.4	164.1	183.8	192.2	116.1	104.1	160.2
30	2015	Myanmar	F446I	67.5	169.2	166.5	162.5	164.2	183.9	184.3	110.2	113.2	159.8
31	2014	Myanmar	A676D	67.4	147.6	160.8	157.3	164.2	180.2	198.4	110.1	107.2	159.9
32	2014	Myanmar	A676D	66.2	159.1	163.5	163.5	164.1	180.1	188.3	134.1	89.2	159.7
33	2015	Myanmar	A676D	66.3	160	172.4	160.4	172.4	183.4	200	125.2	97.8	159.7
34	2014	Myanmar	N458Y	73.1	147.7	157.5	160.5	163.8	177.9	198.1	137.1	104.1	160.1
35	2014	Myanmar	N458Y	73.4	160	163.4	151.1	164	180.2	199.8	137.2	107.1	159.7
36	2013	Myanmar	W443C	66.3	147.7	163.4	135.7	166.8	183.7	188.1	131.2	92	159.8
37	2014	Myanmar	S459L	65.8	144.6	163.6	174.7	164	183.9	186.1	119.2	89.2	159.9
38	2014	Myanmar	C469Y	66.2	147.7	175.4	160.5	164	183.9	198.3	116.2	98	159.9
39	2013	Myanmar	G533A	73.6	141.7	157.5	163.5	166.9	180.1	192.2	116.2	104	159.9
40	2012	Myanmar	P574L	67.4	159.9	166.5	159.4	163.9	177.9	200.1	125.3	95.2	159.9
41	2013	Myanmar	A578S	75.6	160.1	157.5	174.7	164.1	180.2	192.3	116.1	107.2	159.9
42	2013	Myanmar	V692F	66.2	184.3	166.6	166.5	164	242.1	200.2	134.1	107.1	159.9
43	2015	Laos	V581I	72.2	141.6	172.4	157.3	167	180.2	198	134.2	110.2	159.8
44	2012	Tanzania	WT	64.5	141.5	187.1	148.1	164.1	180	192	116.2	107.1	160
45	2013	Ghana	WT	64.1	184.3	160.5	166.5	156.3	180.2	200.1	125.1	95.1	160.1
46	2013	Ghana	WT	72.8	160	160.5	187.8	178.9	242.1	192.1	134.2	95.2	160.1
47	2014	Ghana	WT	64.3	141.7	163.5	166.5	181.8	180.3	183.8	134.1	95.1	160.2
48	2015	Carbon	WT	64.1	169.2	163.4	165.9	181.9	183.8	200.1	110.2	89.4	165.7
49	2017	Cameroon	WT	66.8	159.9	160.4	187.9	178.8	184.5	200.1	137.2	95.2	159.8
50	2013	Ghana	A578S	64.3	160	157.5	157.3	164	242.3	200.3	125.2	113.2	160

51	2017	Ghana	A578S	73.5	159.9	166.5	160.3	166.8	180.1	197.8	125.2	113.1	160
52	2015	Ghana	A578S	65.2	184.5	157.6	174.6	164	242.1	198.2	134.2	107.2	160.2
53	2017	Ghana	L440L	67.1	160	187.1	165.4	167	183.8	198.3	125.2	98	165.8
54	2014	Ghana	D584E	64.2	141.5	163.5	163.5	181.9	180.2	192.2	125.1	86.2	165.8
55	2015	Ghana	A675V	65.1	159.8	166.6	160.5	166.3	183.8	200.1	134.2	98	172.2
56	2016	Ghana	W702C	72.4	184.3	166.5	187.8	166.8	184.4	200	125.1	113.1	159.8
57	2017	Cameroon	C469C	63.2	141.7	166.5	163.5	166.8	180.1	192.1	134.2	98.2	172.2
58	2016	DRC[a]	C469C	66.5	159.9	172.6	157.2	181.9	242.2	198	134.2	98.1	159.8
59	2017	EG[b]	Y500Y	66.1	160	157.6	157.3	166.9	177.9	192.2	125	104.2	172.1
60	2015	SL[c]	T522G	73.6	141.7	166.6	174.5	166.9	180.2	198.2	125.1	107.2	165.9

[a] DRC: The Democratic Republic of the Congo
[b] EG: Equatorial Guinea
[c] SL: Sierra Leone
