# Supplementary material for: Investigation and Evaluation of Genetic Diversity of Plasmodium falciparum Kelch 13 Polymorphisms Imported From Southeast Asia and Africa in Southern China
Source: Front Public Health. 2019 Apr 24;7:95. doi: 10.3389/fpubh.2019.00095 (PMC6491575; doi:10.3389/fpubh.2019.00095)
Supplement: Supplementary file 2 [file Image_1.pdf]

Figure S1 The gel picture for 285 samples in the study. C represents positive control, NTC represents negative control. DL2000 is the molecular marker.

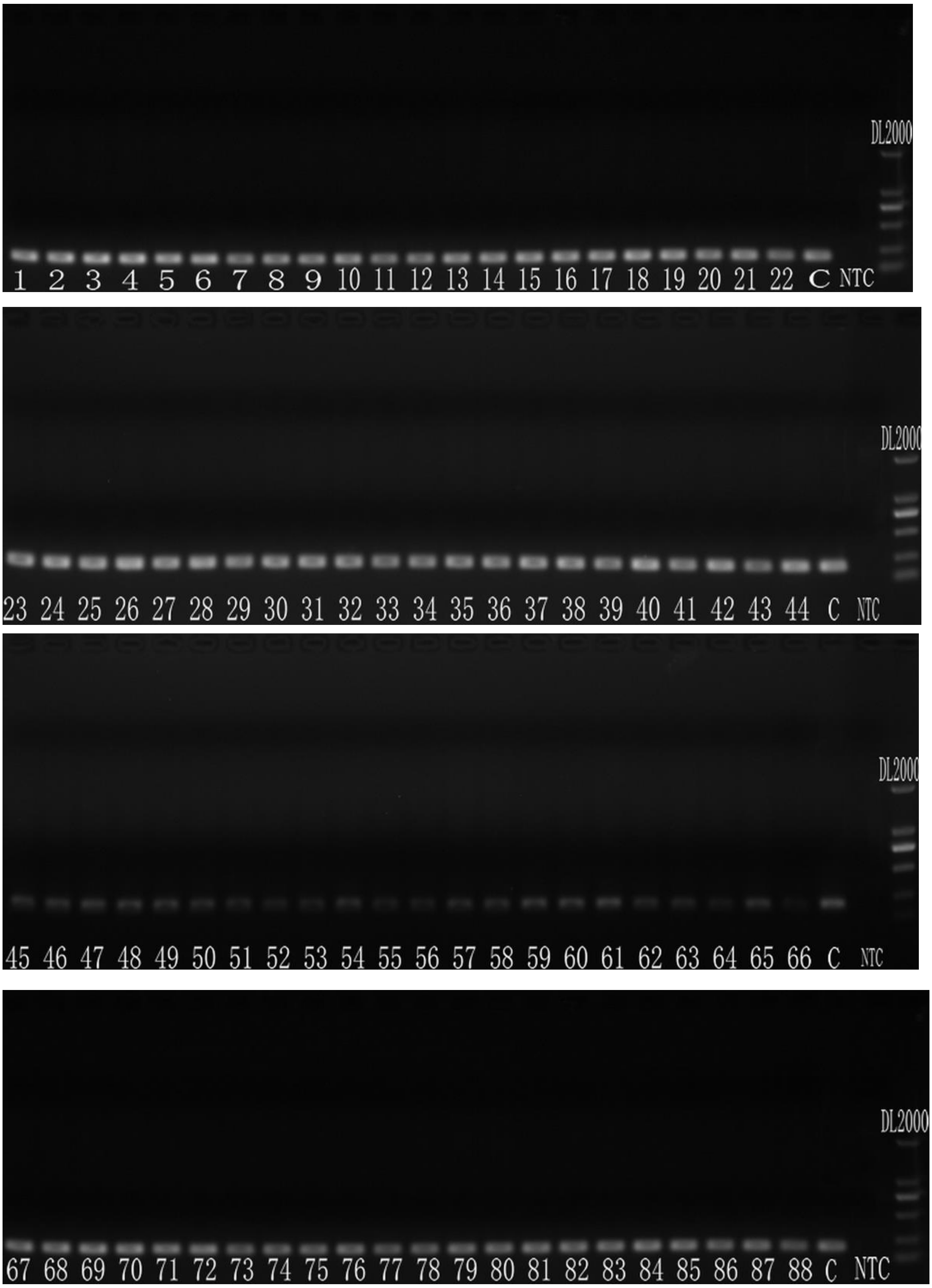

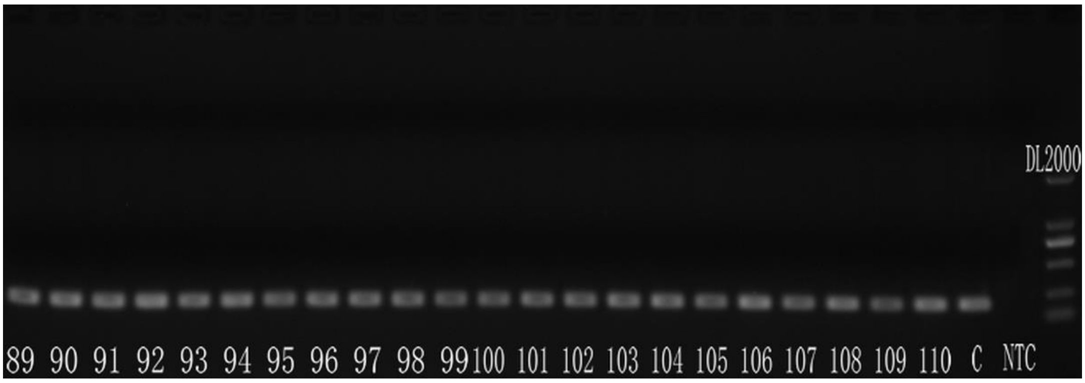
DL2000
89 90 91 92 93 94 95 96 97 98 99 100 101 102 103 104 105 106 107 108 109 110 C NTC

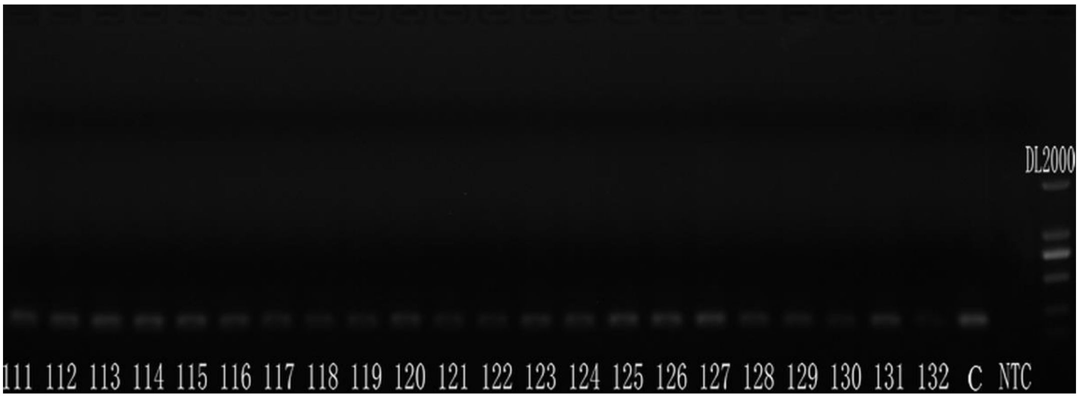
DL2000
111 112 113 114 115 116 117 118 119 120 121 122 123 124 125 126 127 128 129 130 131 132 C NTC

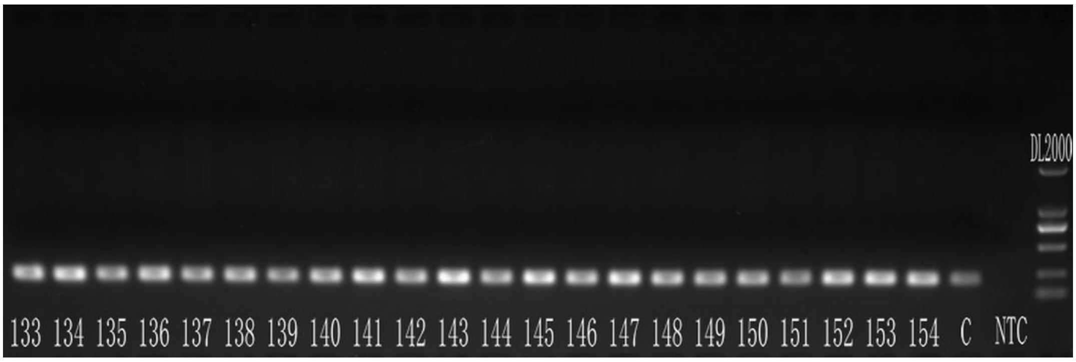
DL2000
133 134 135 136 137 138 139 140 141 142 143 144 145 146 147 148 149 150 151 152 153 154 C NTC

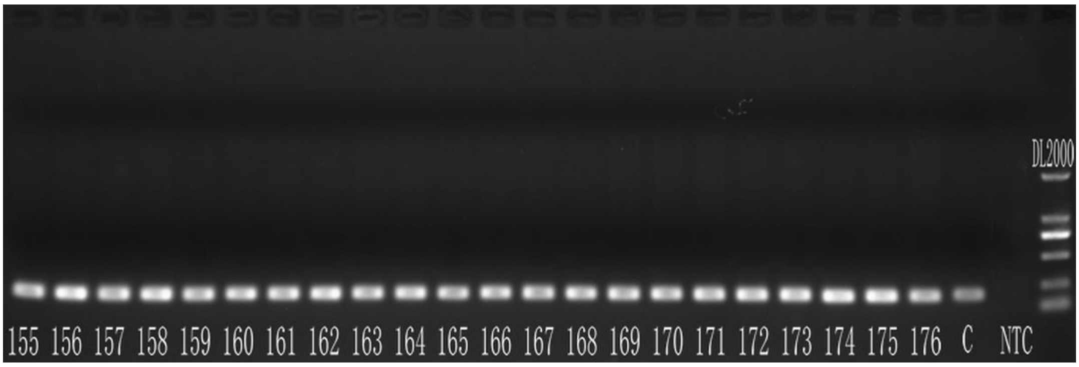
DL2000
155 156 157 158 159 160 161 162 163 164 165 166 167 168 169 170 171 172 173 174 175 176 C NTC

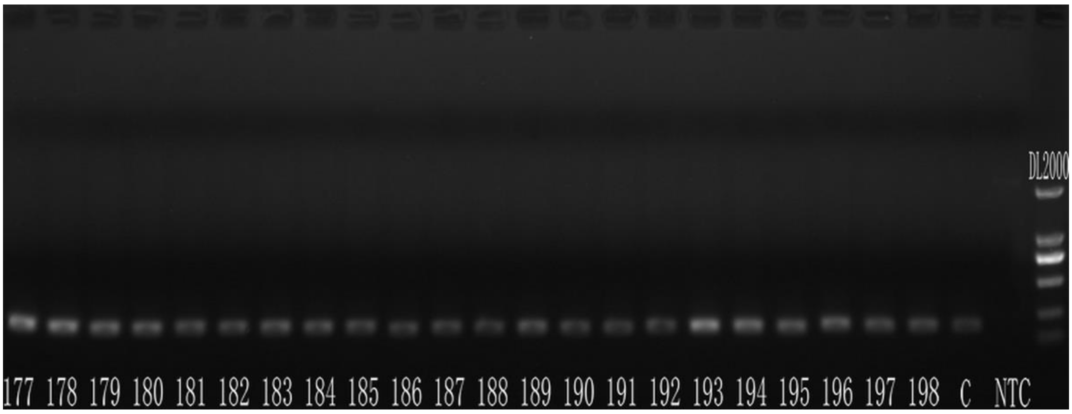
DL2000
177 178 179 180 181 182 183 184 185 186 187 188 189 190 191 192 193 194 195 196 197 198 C NTC

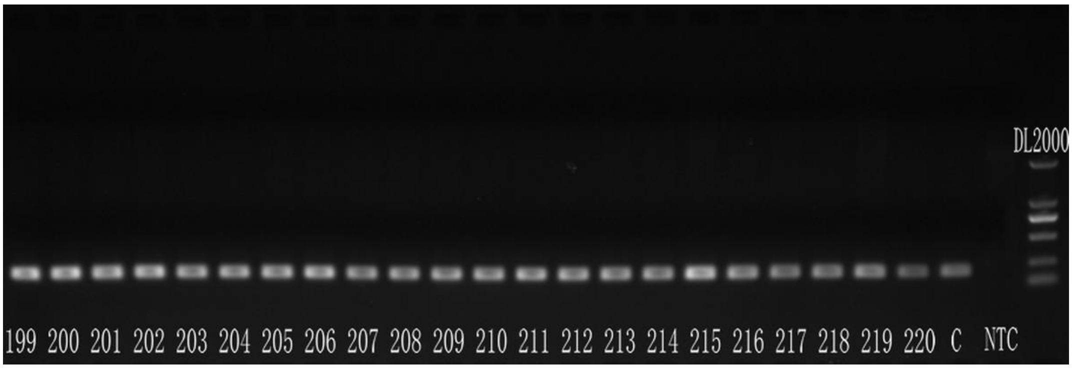
DL2000
199 200 201 202 203 204 205 206 207 208 209 210 211 212 213 214 215 216 217 218 219 220 C NTC

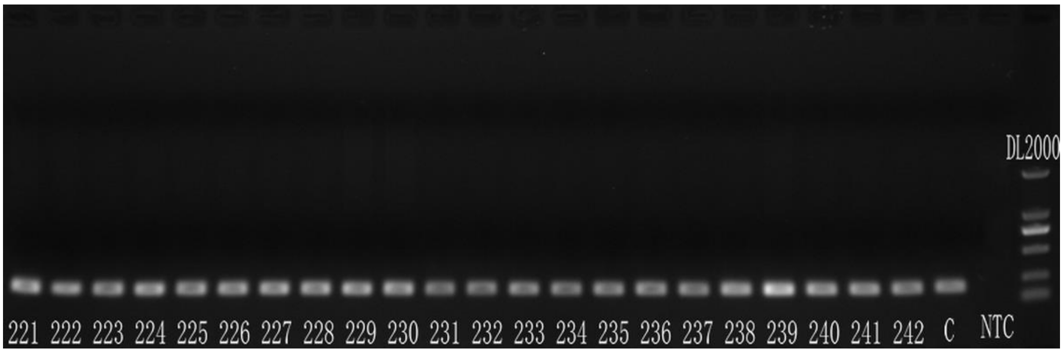
DL2000
221 222 223 224 225 226 227 228 229 230 231 232 233 234 235 236 237 238 239 240 241 242 C NTC

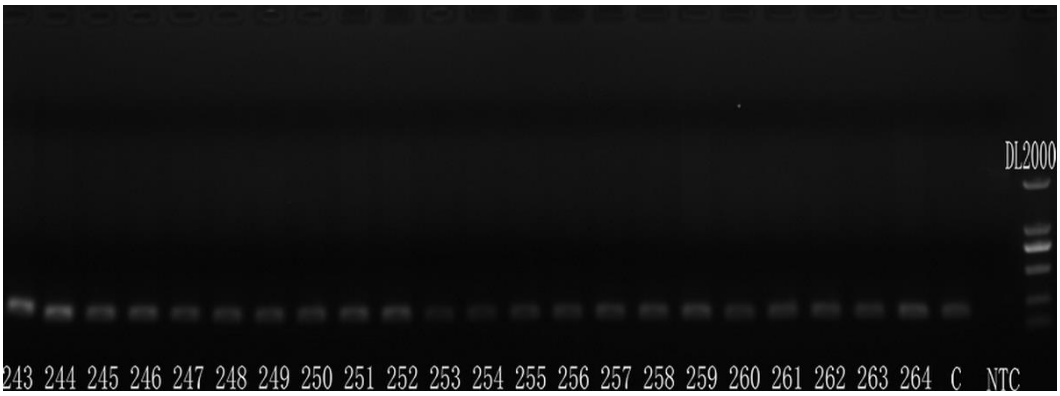
DL2000
243 244 245 246 247 248 249 250 251 252 253 254 255 256 257 258 259 260 261 262 263 264 C NTC

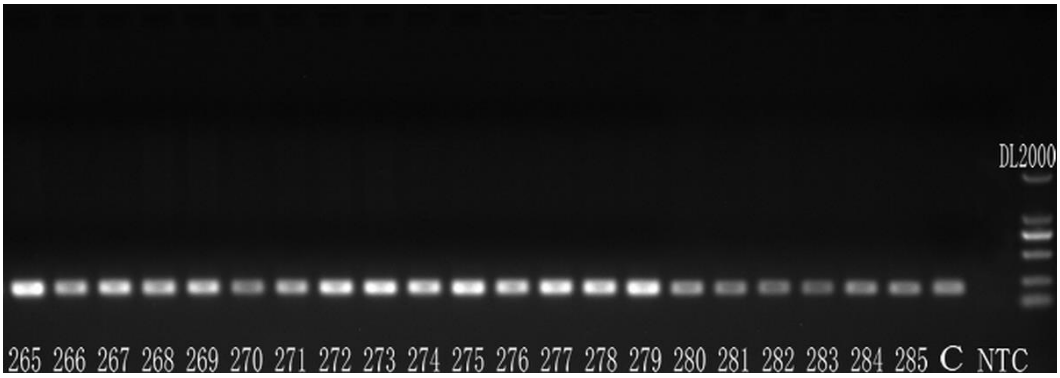
DL2000
265 266 267 268 269 270 271 272 273 274 275 276 277 278 279 280 281 282 283 284 285 C NTC
